# *Corynebacterium glutamicum* possesses β-N-acetylglucosaminidase

**DOI:** 10.1186/s12866-016-0795-3

**Published:** 2016-08-05

**Authors:** Christian Matano, Stephan Kolkenbrock, Stefanie N. Hamer, Elvira Sgobba, Bruno M. Moerschbacher, Volker F. Wendisch

**Affiliations:** 1 Genetics of Prokaryotes, Faculty of Biology & CeBiTec, Bielefeld University, 33501 Bielefeld, Germany; 2 Institute for Biology and Biotechnology of Plants, WWU Münster University, 48143 Münster, Germany; 3 Present Address: GSK Vaccines S.r.l., Siena, 53100 Italy; 4 Present address: altona Diagnostics GmbH, 22767 Hamburg, Germany

**Keywords:** NagA_2_, N-acetylglucosaminidase, *Corynebacterium glutamicum*, Secretion, Chitinase

## Abstract

**Background:**

In Gram-positive *Corynebacterium glutamicum* and other members of the suborder Corynebacterianeae, which includes mycobacteria, cell elongation and peptidoglycan biosynthesis is mainly due to polar growth. *C. glutamicum* lacks an uptake system for the peptidoglycan constituent N-acetylglucosamine (GlcNAc), but is able to catabolize GlcNAc-6-phosphate. Due to its importance in white biotechnology and in order to ensure more sustainable processes based on non-food renewables and to reduce feedstock costs, *C. glutamicum* strains have previously been engineered to produce amino acids from GlcNAc. GlcNAc also is a constituent of chitin, but it is unknown if *C. glutamicum* possesses chitinolytic enzymes.

**Results:**

Chitin was shown here not to be growth substrate for *C. glutamicum.* However, its genome encodes a putative N-acetylglucosaminidase. The *nagA*_*2*_ gene product was active as β-N-acetylglucosaminidase with 0.27 mM 4-nitrophenyl N,N’-diacetyl-β-D-chitobioside as substrate supporting half-maximal activity. NagA2 was secreted into the culture medium when overproduced with TAT and Sec dependent signal peptides, while it remained cytoplasmic when overproduced without signal peptide. Heterologous expression of exochitinase gene *chiB* from *Serratia marcescens* resulted in chitinolytic activity and ChiB secretion was enhanced when a signal peptide from *C. glutamicum* was used. Colloidal chitin did not support growth of a strain secreting exochitinase ChiB and β-N-acetylglucosaminidase NagA2.

**Conclusions:**

*C. glutamicum* possesses β-N-acetylglucosaminidase. In the wild type, β-N-acetylglucosaminidase activity was too low to be detected. However, overproduction of the enzyme fused to TAT or Sec signal peptides led to secretion of active β-N-acetylglucosaminidase. The finding that concomitant secretion of endogenous NagA2 and exochitinase ChiB from *S. marcescens* did not entail growth with colloidal chitin as sole or combined carbon source, may indicate the requirement for higher or additional enzyme activities such as processive chitinase or endochitinase activities.

**Electronic supplementary material:**

The online version of this article (doi:10.1186/s12866-016-0795-3) contains supplementary material, which is available to authorized users.

## Background

Corynebacteria are Gram-positive microorganisms closely related to mycobacteria. *C. glutamicum* is considered a model organism for other members of the suborder Corynebacterianeae. Likewise, *C. glutamicum* is a model bacterium for white biotechnology since it is used for the production of amino acids and derived products [1]. This bacterium can use a variety of carbon sources for growth and production, e.g. sugars (glucose, fructose, sucrose, maltose, ribose), alcohols (ethanol, myo-inositol) and organic acids (acetate, propionate, D- or L-lactate, gluconate, pyruvate) [2–6]. Metabolic engineering has been applied to *C. glutamicum* aimed at improving amino acid production, to enable production of novel compounds such as diamines [7, 8] or terpenoids [9–11], and to enable a flexible feedstock concept [12]. Feedstock costs are an important part of the overall fermentation costs and access to a wide range of carbon substrates, in particular non-food renewables, is sought [2]. Recombinant strains of *C. glutamicum* which can utilize glycerol [13, 14], starch [15, 16], arabinose and xylose derived from lignocellulosic biomass [17–21] or the amino sugars glucosamine (GlcN) and N-acetyl-glucosamine (GlcNAc) have been developed [22, 23]. GlcN and GlcNAc represent interesting feedstocks for biotechnological applications since they can be produced by acid hydrolysis of chitin, one of the major waste components generated from shellfish industry [24, 25]. Shrimp farming is a continuously growing sector concentrated in Southeast Asia and China, where every year around 4 million MT of shrimps are harvested, with a chitin-containing waste generation of around 40 % of shrimp dry weight [26, 27]. Thus, the amino sugar fraction of shellfish waste could represent an example of a sustainable, renewable feedstock for industrial fermentations, in particular for nitrogenous compounds such as amino acids and diamines.

*C. glutamicum* lacks the transporter for GlcNAc so that the heterologous overexpression of a specific transporter is necessary in order to achieve growth on this carbon source [22]. GlcNAc-6P is generated intracellularly during catabolism of N-acetylneuramic acid (Neu5Ac), a carbon source for *C. glutamicum*. The sialic acid transport and utilization genes are clustered in *C. glutamicum* ATCC 13032 together with a regulator (cg2936) belonging to the GntR-family transcription factor (in the region cg2929-cg2940) [28, 29]. The genome of *C. glutamicum* ATCC 13032 contains a gene (cg3158, *nagA*_*2*_) annotated to encode a putative β-N-acetylglucosaminidase precursor, whose activity and function has not yet been studied. β-N-acetylglucosaminidases (E.C. 3.2.1.52) are characterized by the ability to cleave off single units of GlcNAc from the non-reducing ends of short oligosaccharides or peptidoglycan. A common N-acetylglucosaminidase of bacteria is NagZ, a GH3 family hydrolase that participates in bacterial cell wall recycling [30]. In the Gram-negative model organism *E. coli*, NagZ is located in the cytoplasm and removes GlcNAc from 1,6-anhydroMurNAc-peptides, that derive from the breakdown of the murein envelope by transglycosylases and endopeptidases during growth [31]. In many Gram-negative bacteria, cell wall recycling is also linked to antibiotic response: the organisms can detect β-lactam antibiotics affecting cell wall formation by sensing an increase in intracellular muropeptides concentration, eliciting resistance mechanisms [30].

Whether Gram-positive bacteria recycle their cell wall has been questioned for a long time, but recently putative cell recycling pathways have been elucidated for *Bacillus subtilis* and *Clostridium acetobutylicum* [32, 33]. In contrast to *E. coli* and *C. acetobutylicum*, muropeptide cleavage occurs extracellularly in *B. subtilis*, whose NagZ is secreted to the medium, where it hydrolyzes GlcNAc from muropeptide units. The released products are transported into the cytoplasm by specific phosphotransferases and further processed for recycling or energy production [34]. In each generation, up to 50 % of peptidoglycan is turned over in *B. subtilis* and *E. coli* [35], due to their cell division mechanism, characterized by intercalation of new cell wall along most of their length. To our knowledge, it is not known whether *C. glutamicum* recycles peptidoglycan, given that the apical cell elongation occurring during its cell division may not require a massive cell wall breakdown [36, 37]. *C. glutamicum* lacks orthologs of most of the genes responsible for peptidoglycan recycling as present in *E. coli* [35]. Moreover, since *C. glutamicum* does not possess a specific transporter for GlcNAc, it cannot import and utilize GlcNAc as carbon source unless a gene for GlcNAc transport is expressed heterologously [22].

N-acetylglucosaminidase also catalyzes the last step of bacterial chitin degradation. Many chitinolytic organisms produce a cocktail of chitinases that hydrolyze chitin to the disaccharide N,N'-diacetyl chitobiose as major end product, which is eventually cleaved by a chitobiosidase to GlcNAc monomers [38]. GlcNAc production by enzymatic treatment using specific enzymes from chitinolytic organisms and unspecific crude enzymes such as lysozyme, papaine or lipase is seen more favorably than chemical hydrolysis, since enzymatic hydrolysis of chitin operates under mild conditions without generating large amounts of wastes, an environmental concern of the chemical method [24, 39].

Here we report that *C. glutamicum* possesses N-acetylglucosaminidase activity and that the respective enzyme is encoded by cg3158/*nagA*_*2*_. Its activity was compared to that of the already characterized NagZ from *B. subtilis*. Furthermore, variants of NagA_2_, fused to different tags for secretion or without secretion signal, were constructed and tested. The variants with higher secreted activity have been combined in a plasmid with a secreted chitinase, ChiB, from the chitinolytic Gram-negative *Serratia marcescens*, in order to test if the combination of chitinase and N-acetylglucosaminidase activity supports the growth of *C. glutamicum* with chitin as carbon source.

## Results

### *C. glutamicum* ATCC13032 possesses a GH3 N-acetylglucosaminidase

**Fig. 1:**
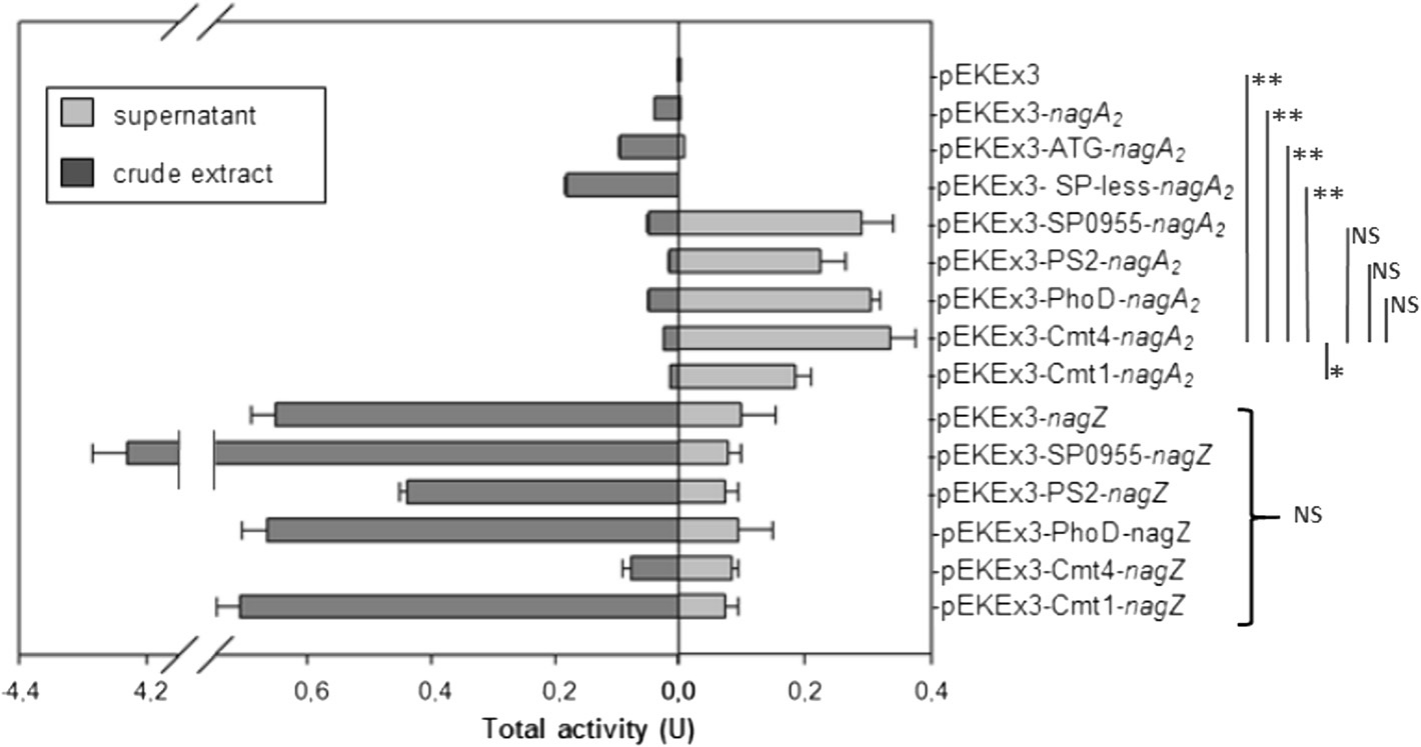
N-Acetylglucosaminidase activities in supernatants (extracellular fraction) and crude extracts (cellular fraction) from cultures of *C. glutamicum* Δ*nanR* transformed with the indicated plasmids. Data represent means and SD from activity assays conducted on three independent cultivations. All results were tested for significance using the paired heteroscedastic Student’s t-test. The level of significance of the differences observed between the control and test samples was expressed as one, two or three stars, for * *p* ≤ 0.05, and ** *p* ≤ 0.01, respectively. “NS” stands for nonsignificant

BLAST alignments indicate that *nagA*_*2*_ encodes a β N-acetylglucosaminidase belonging to the family 3 of glycosidases, which is predicted to be secreted. As other GH3 family β N-acetylglucosaminidases characterized so far, NagA_2_ possesses the highly conserved sequence motif KHFPGHGX(4) **D**S**H**, where the aspartyl and histidyl residues (indicated in bold) form a catalytic dyad as elucidated for the first time for *B. subtilis* NagZ [40] (Table 1). The gene *nagA*_*2*_ was cloned in an overexpression vector in order to determine whether it encodes for an active protein. An additional variant of *nagA*_*2*_ was cloned, replacing the translational start codon GTG with ATG, the preferred start codon of *C. glutamicum* [41]. Overexpression of the native gene (GTG start codon) led to detectable N-acetylglucosaminidase activity. While most of the activity was found in the cytoplasmic fraction, about one third of the activity was in the supernatant. Replacing the start codon of the *nagA*_*2*_ gene with ATG led to a two fold increase of the activity both in the cell extract (specific activity of 3.7 ± 0.2 mU mg^−1^, corresponding to a total activity 0.09 U in a grown 50 mL culture) and in the supernatant fraction (total activity of 0.01 U in the 50 mL broth). By contrast, no extracellular activity was detected upon overexpression of a *nagA*_*2*_ version lacking the predicted signal peptide, while the intracellular specific activity reached the highest value of 5.4 ± 0.3 mU mg^−1^, corresponding to about 0.18 U in a grown 50 mL culture (Fig. 1). The overall activity is relatively low when compared to the heterologous overexpression of *nagZ* from *B. subtilis* (Fig. 1). NagZ produced in *C. glutamicum* led to an intracellular specific activity of N-acetylglucosaminidase of 40.7 ± 4.8 mU mg^−1^ (circa 0.6 U in the 50 mL broth), more than 25 fold higher than for the corresponding *C. glutamicum* native enzyme. Taken together, the results indicated that *nagA*_*2*_ encodes a secreted N-acetylglucosaminidase, although secretion was not efficient.

**Table 1:**
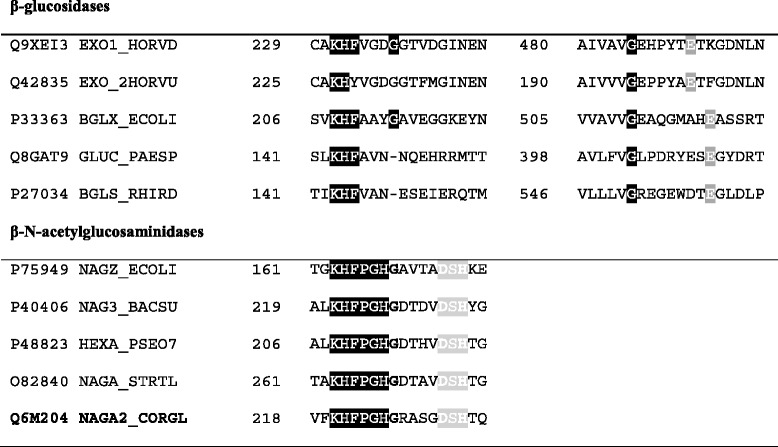
Partial sequence alignment of selected β-N-acetylglucosaminidases and β-glucosidases from glycosyl hydrolases family 3

The subfamilies of glycosyl hydrolases family 3 are distinguished by the sequence pattern next to the conserved KH (F/Y) motif (black boxed letters) at N-terminal domain adjacent to β-strand 5 of the N-terminal (β/α)_8_ barrel domain. The residues involved in the catalytic mechanism are boxed in light grey: while in β-glucosidases, a glutamate at a C-terminal region is responsible for the acid/base catalytic mechanism, N-acetylglucosaminidases are characterized by the D (S/T)H motif containing the Asp-His catalytic dyad [40]. Q9XEI3 and Q42835 are β-D-glucan exohydrolases from *Hordeum vulgare*, P33363 is BglX from *E. coli*, Q8GAT9 glucocerebrosidase from *Paenibacillus* sp. T12, P27034 is cellobiase from *Rhizobium radiobacter*, P75949 is Nag Z from *E. coli*, P40406 is NagZ from *B. subtilis* str. 168, P48823 is Cht60 from *Pseudoalteromonas piscicida*, O82840 is NagA from *Streptomyces thermoviolaceus*, Q6M204 is putative β-N-acetylglucosaminidase NagA_2_ from *C. glutamicum* ATCC13032

### Overexpression of *nagA*_*2*_ fused with homologous N-terminal signal peptides increased extracellular activity of the enzyme

In order to increase the secretion of the enzyme, the putative signal peptide of *nagA*_*2*_ was replaced by various endogenous signal peptides known to enable secretion in *C. glutamicum* [42–46]. Different TAT and Sec pathway signal peptides were considered in this work (Table 2): SP0955, a TAT secretion signal homologous to *C. glutamicum* R cgR_0949 [42, 43]; PS2, a Sec-pathway signal peptide of *C. glutamicum* ATCC 14067 PS2 protein, which is missing in *C. glutamicum* ATCC 13032 [44, 45]; PhoD, a TAT-pathway signal peptide from *cg2485* encoding secreted alkaline phosphatase [46]; Cmt4 and Cmt1, Sec pathway secretion signals of *cg2394* coding for corynomycolyl transferase, and, *cg0413* coding for trehalose corynomycolyl transferase, respectively [43].

**Table 2:** Protein coding sequences exhibiting Tat-type signal sequences in *C. glutamicum*

Protein ID, locus tag	Annotation	Signal sequence^a^	Length (aa)	Secretion pathway
Cg0955, *cg0955*	secreted protein	MQINRRGFLKATTGLATIGAASMFMPK**ANA**/**LG**	30	TAT
PS2, *cgR_2070*	S-layer protein	MGKHRRNNSNATRKAVAASAVALGATAAIASP**AQA**/**AE**	35	Sec
PhoD, *cg2485*	alkaline Phosphatase	MPQLSRRQFLQTTAVTAGLATFAGTP**ARA**/**EE**	29	TAT
Cmt4, *cg2394*	corynomycolyl transferase	MRKGISRVLSVAVASSIGFGTVLTGTGI**AAA**/**QD**	31	Sec
Cmt1, *cg0413*	trehalose corynomycolyl transferase	MKLLRRIAAPAIALGIAMSTIVTPST**AGA**/**AE**	29	Sec

^a^The amino acids residues in bold indicate residues involved in signal peptide cleavage and the putative cleavage site is marked by a solidus (/)

Both, the overall activity of NagA_2_ and its secretion efficiency, greatly improved upon replacing the native secretion signal peptide. N-acetylglucosaminidase activity in the supernatant ranged from about 85 % to 93 % of the total activity measured inside the cells and in the broth. The signal peptides SP0955, PhoD and Cmt4 led to the highest enzyme activities in the culture broth (corresponding to 0.29 ± 0.05 U, 0.31 ± 0.02 U and 0.34 ± 0.04 U, respectively).

Thus, about sixty fold more N-acetylglucosaminidase activity as compared to the native enzyme was found in the supernatant (Fig. 1). Signal peptides are cleaved during protein export and are expected not to affect kinetic parameters of the mature enzymes. Indeed, the K_m_ values of NagA_2_ lacking its own signal peptide (0.266 ± 0.037 mM) or fused to the *C. glutamicum* PhoD signal peptide (0.270 ± 0.066 mM) were shown to be comparable.

*B. subtilis* NagZ was also expressed with various signal peptides in *C. glutamicum* (Fig. 1). However, fusion to endogenous signal peptides did not increase the amount of enzyme activity retrieved in the supernatant. Notably, fusion to the signal peptide from Cg0955 increased intracellular enzyme activity, whereas fusion to signal peptide Cmt4 led to reduced intracellular N-acetylglucosaminidase activity (Fig. 1).

### Overexpression of ChiB fused with a secretion signal peptide from *C. glutamicum* enabled secretion of the enzyme

**Fig. 2:**
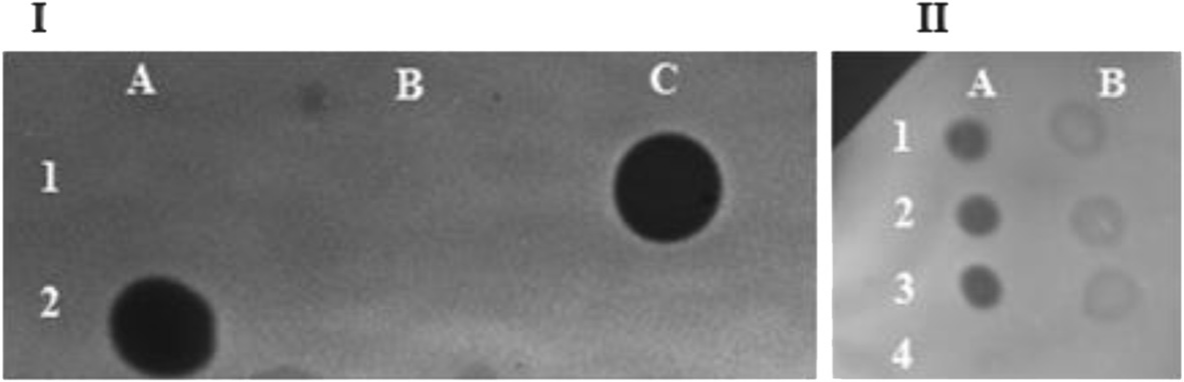
Dot activity assay on glycol chitin gels to detect chitinase activity. **I** Five μL of cell extract (A) and supernatant (B) from *C. glutamicum* WT (pEKEx3) (1) and *C. glutamicum* WT (pEKEx3-*chiB*) (2) are spotted on the glycol chitin gel. One μL of purified ChiB from *S. marcescens* with a specific activity of 28.8 mU mL^−1^ was spotted as a positive control (C1). ChiB activity is clearly seen in the cell extract of *C. glutamicum* overexpressing ChiB, while no activity is detected on the supernatant of this strain or in the wildtype. **II** Triplicates (1–3) of one μL of concentrated (Amicon 30 K) culture supernatant (A) and 5 μL of non-concentrated supernatant (B) of *C. glutamicum* WT (pEKEx3-SP0955-*chiB*) and the empty vector control *C. glutamicum* WT (pEKEx3) (A4) were spotted on the glycol chitin gel

The heterologous overexpression of *chiB* from *S. marcescens* in *C. glutamicum* resulted in the accumulation of an active product in the cytoplasm. No extracellular activity was detected in a glycol chitin gel assay, indicating that the *chiB* signal peptide from *S. marcescens* is not recognized by the host (Fig. 2-I). Secretion was achieved by fusing the sequence encoding the TAT secretion signal peptide from the *C. glutamicum* gene *cg0955* to *chiB,* and activity was detectable on the glycol chitin gel (Fig. 2-II). Subsequently, chitinase activity was quantified by measuring the hydrolysis of p-nitrophenol from 4-Nitrophenyl N,N’-diacetyl-β-D-chitobioside. *C. glutamicum* strain Δ*nanR* (pEKEx3*-*SP0955*-chiB*) revealed total chitinase activity of about 0.33 U in the 50 mL culture supernatant. ChiB fusion with the signal peptide from Cg0955 not only allowed secretion, but also increased the specific intracellular activity about eight fold from 0.5 ± 0.1 mU mg^−1^ for native *chi*B to 3.8 ± 0.3 mU mg^−1^ (Table 3).

**Table 3:** Chitinase activity in the cellular and extracellular fraction of *C. glutamicum* Δ*nanR* strain overexpressing *chiB* from *S. marcescens* in its native form or fused with the signal peptide from Cg0955

	Chitinase activity		
	Intracellular		Extracellular
Strain	sp. act. (mU/mg)	total activity (U)	total activity (U)
Δ*nanR* (pVWEx1-*nagE*) (pEKEx3)	<0.01	<0.001	<0.01
Δ*nanR* (pEKEx3-*chiB*)	0.5 ± 0.1	0.14 ± 0.05	<0.01
Δ*nanR* (pEKEx3-*SP0955-chiB*)	3.8 ± 0.3	0.17 ± 0.01	0.22 ± 0.03

Data represent means and SD from activity assays conducted on three independent cultivations

### Concomitant overproduction of ChiB and NagA_2_ is not sufficient to support growth with colloidal chitin in a *C. glutamicum* strain able to grow with GlcNAc

**Fig. 3:**
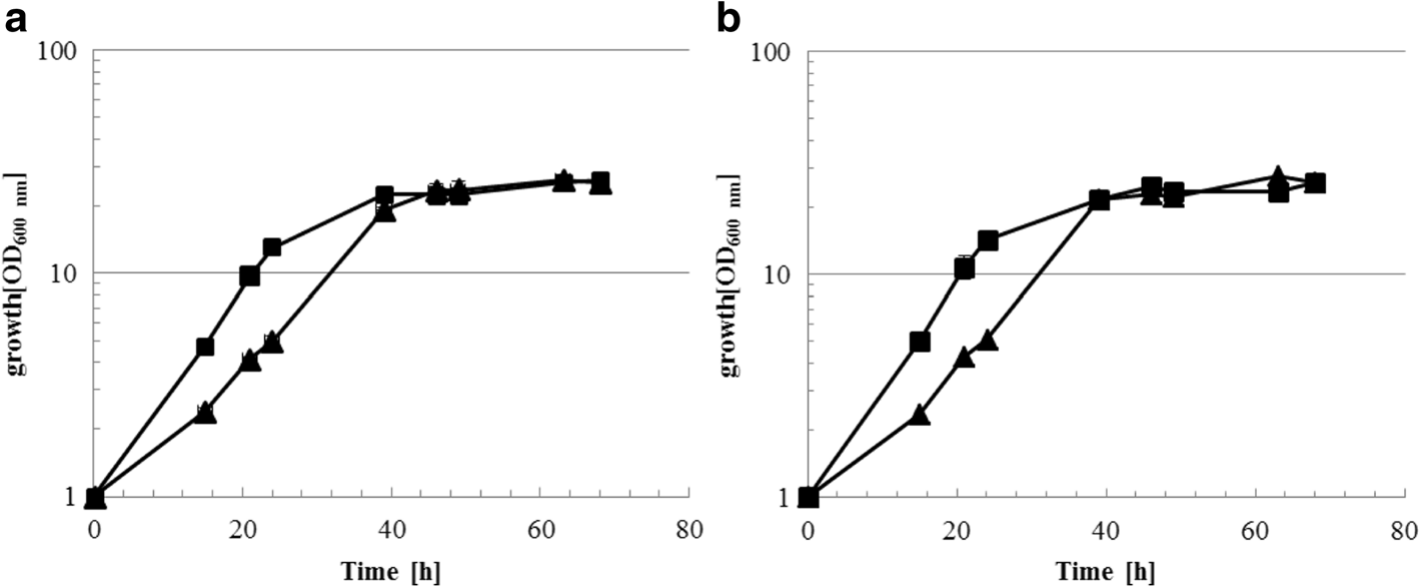
Growth of *C. glutamicum* Δ*nan*R (pVWEx1-*nagE*) (pEKEx3) [triangles] and Δ*nan*R (pVWEx1-*nagE*) (pEKEx3-SP0955-*chi*B-SP0955-*nagA*_*2*_) [squares] in CgXII minimal medium with 100 mM GlcNAc (**a**) or 100 mM GlcNAc and 1 % colloidal chitin (**b**) as carbon and energy sources. Data represent means and SD from three independent cultivations

As reported previously, utilization of GlcNAc as substrate in *C. glutamicum* requires a heterologous GlcNAc uptake system (e.g. NagE from *C. glycinophilum*) in addition to high NagA and NagB activities [22]. Overexpression of both *nagA* and *nagB* is possible either from a plasmid [22] or by derepression via deletion of the GntR-family transcription factor gene *nanR*, present in the vicinity of the *nagAB-scrB* operon (Matano & Wendisch, unpublished). In this study, *C. glutamicum* Δ*nanR* (pVWEx1-*nagE*) was transformed with vectors for combined overexpression of endogenous *nagA*_*2*_ and of *chiB* from *S. marcescens* (plasmids maps are depicted in Additional file 1: Figure S1)*.* The *chiB* gene was cloned with the signal peptide SP0955 and *nagA*_*2*_ was cloned with the signal peptides SP0955, PhoD or Cmt4. The resulting strains showed a total ChiB activity in the supernatants of around 0.20 U (Table 4). With respect to N-acetylglucosaminidase activities, the strain producing SP0955-*nagA*_*2*_ showed the highest activity (0.17 ± 0.02 U) as compared to PhoD-*nagA*_*2*_ (0.08 ± 0.00 U) and Cmt4-*nagA*_*2*_ (0.11 ± 0.0 U) (Table 4). Therefore, *C. glutamicum* strain Δ*nanR* (pVWEx1-*nagE*) (pEKEx3-SP0955-*chiB*-SP0955-*nagA*_*2*_) was chosen for growth experiments with colloidal chitin as sole carbon source. While this strain grew with GlcNAc as sole carbon source (Fig. 3a), no growth with colloidal chitin as sole carbon source was observed (data not shown). With minimal medium containing a mixture of 100 mM GlcNAc and 1 % (w/v) colloidal chitin as carbon sources, strain Δ*nanR* (pVWEx1-*nagE*) (pEKEx3-SP0955-*chiB*-SP0955-*nagA*_*2*_) grew to a comparable final OD (25.7 ± 2.6) as with GlcNAc as sole carbon source (final OD of 26.4 ± 0.6) (Fig. 3b). The maximal growth rate was 0.11 ± 0.1 h^−1^ under both conditions. The control strain Δ*nanR* (pVWEx1-*nagE*) reached a comparable final OD (25.7 ± 1.1) in both media, but grew a little slower with a maximal growth rates of 0.085 ± 0.01 h^−1^ with GlcNAc and 0.88 ± 0.01 h^−1^ with GlcNAc plus colloidal chitin (Fig. 3).

**Table 4:** Chitinase and β-N-acetylglucosaminidase activities in the strain overexpressing SP0955-*chiB* and *nagA*_*2*_ variants with different secretion tags

*C. glutamicum* strain	ChiB activity			NagA2 activity		
	Intracellular		Extracellular	Intracellular		Extracellular
Δ*nanR* (pVWEx1-*nagE*) carrying vector	sp. act. (mU/mg)	Total activity (U)	Total activity (U)	sp. act. (mU/mg)	Total activity (U)	Total activity (U)
pEKEx3-SP0955*-chiB-*SP0955-*nagA*_*2*_	8.7 ± 0.7	0.22 ± 0.01	0.22 ± 0.03	2.1 ± 0.2	0.05 ± 0.01	0.17 ± 0.02
pEKEx3-SP0955-*chiB*-PhoD-*nagA*_*2*_	8.1 ± 0.8	0.20 ± 0.01	0.21 ± 0.01	2.4 ± 0.3	0.06 ± 0.01	0.08 ± 0.01
pEKEx3- SP0955-*chiB*-Cmt4-*nagA*_*2*_	7.3 ± 0.3	0.16 ± 0.01	0.13 ± 0.01	0.7 ± 0.1	0.02 ± 0.01	0.11 ± 0.01

Data represent means and SD from activity assays conducted on three independent cultivations

**Fig. 4:**
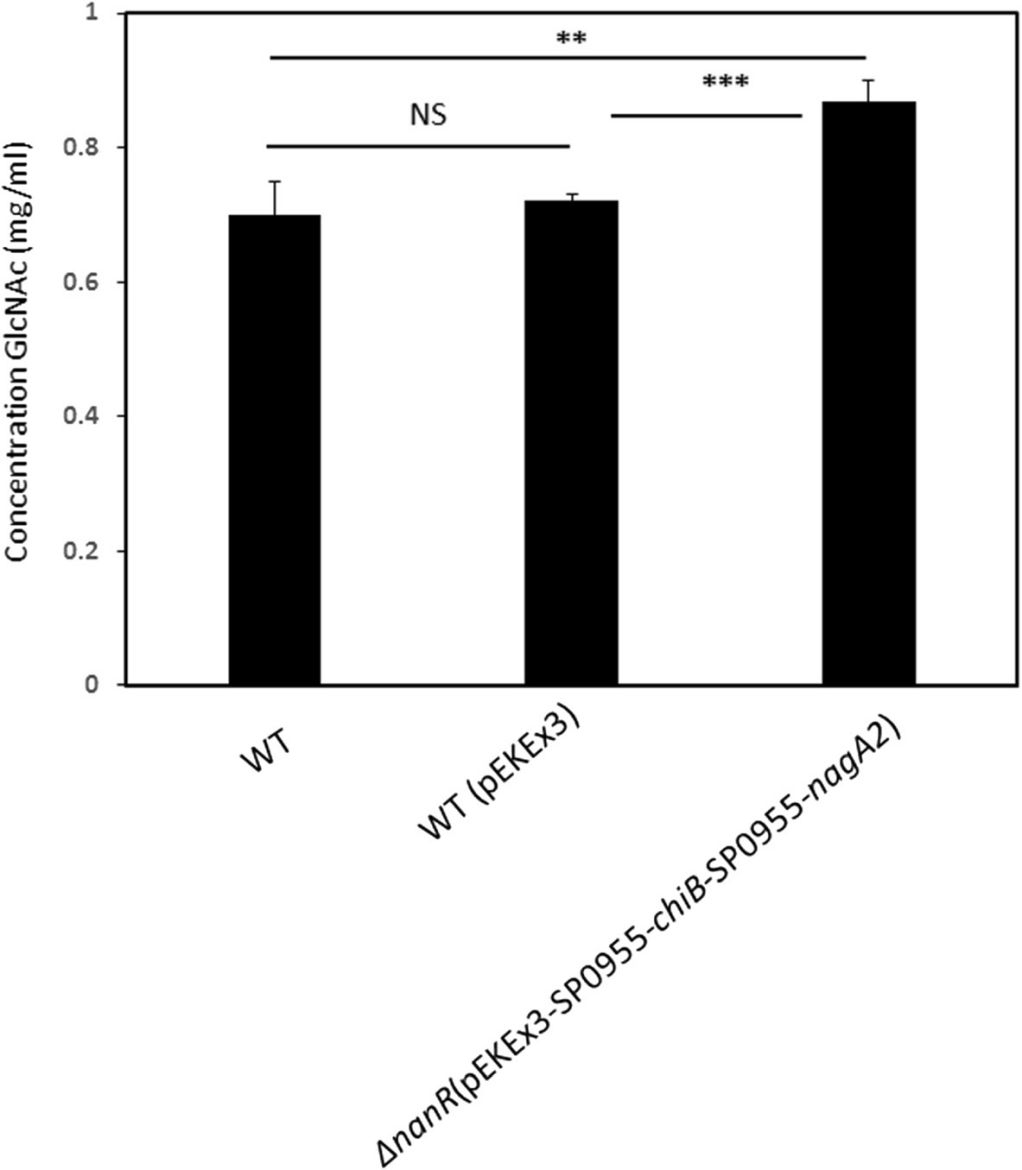
Quantification of GlcNAc in culture supernatant by DNS colorimetric assay in WT, WT (pEKEx3) and ∆*nanR* (pEKEx3-SP0955-*chiB*-SP0955-*nagA2*). Values and error bars represent the mean and the standard errors of triplicate cultures. All results were tested for significance using the paired heteroscedastic Student’s t-test. The level of significance of the differences observed between the control and test samples was expressed as one, two or three stars, for * *p* ≤ 0.05, ** *p* ≤ 0.01 and *** *p* ≤ 0.001, respectively. “NS” stands for nonsignificant

Thus, although enzyme activities of chitinase ChiB and β-N-acetylglucosaminidase NagA2 could be detected, they might be too low to sustain growth with colloidal chitin. In order to test if indeed colloidal chitin is degraded to GlcNAc, supernatants of strains ∆*nanR* (pEKEx3-SP0955_*chiB*-SP0955_*nagA2*), the control strain WT (pEKEx3), and *C. glutamicum* WT were analysed for reducing sugars by the 3,5-dinotrosalicidic acid (DNS) colorimetric assay after incubation for 72 h in minimal medium with 1 % colloidal chitin. Indeed, the culture supernatant of strain ∆*nanR* (pEKEx3-SP0955_*chiB*-SP0955_*nagA2*) showed a low (0.88 ± 0.08 mM), but significantly (*p* < 0.01) higher concentration of reducing sugars than the supernatants of the control strain WT (pEKEx3) and WT (0.72 ± 0.01 mM and 0.70 ± 0.05 mM, respectively, Fig. 4). Despite of some degree of colloidal degradation, the amount of released GlcNAc appeared insufficient to promote cell growth.

## Discussion

Here, *C. glutamicum* was shown to possess N-acetylglucosaminidase activity that is encoded by cg3158/*nagA*_*2*_. Structurally, the NagA_2_ protein belongs to the family 3 glycoside hydrolases, and among these, the N-acetyl-β-D-glucosaminidases show a selective specificity for GlcNAc as substrate [47] with only few exceptions [48]. N-acetyl-β-D-glucosaminidase activity was assayed with 4-nitrophenyl N,N’-diacetyl-β-D-chitobioside as substrate, and about 0.27 mM supported half-maximal activity. In comparison, NagZ from *E. coli* had a higher K_m_ on the same substrate (0.43 mM) [49], whereas NagZ of *B. subtilis* showed an about two fold lower K_m_ of 0.11 ± 0.0 mM with 4'-methylumbelliferyl-β-GlcNAc as substrate [40].

The role of the NagA_2_ activity in *C. glutamicum* is still unclear. Analysis of the *C. glutamicum* transcriptome revealed that the *nag*A_2_ gene is transcribed as leaderless transcript with a relatively low RNA abundance [41]. It is not known whether *nag*A2 expression is regulated in *C. glutamicum*. The adjacent gene cg3157 encodes a hitherto uncharacterized protein with VanW (pfam04294) and peptidoglycan binding domains (pfam12229) that may play a role in cell wall biosynthesis. In contrast, the orthologous *nag*Z is part of operon in *E. coli* (*hinT-ycfL-lpoB-thiK-nagZ-ycfP*) and *B. subtilis* (*ybbIHFEDC*). NagZ from *B. subtilis* and *E. coli* have been shown to play important roles in cell wall recycling [30, 32, 50]. *C. glutamicum* lacks orthologs for most of the genes responsible for muropeptide recycling in *B. subtilis* and orthologs for *E. coli* genes necessary for import and catabolism of anhydromuropeptides. Therefore, further studies need to be performed in order to elucidate whether *C. glutamicum* has a peptidoglycan recycling mechanism and if NagA_2_ is involved.

The endogenous protein NagA_2_ was shown to be secreted, although inefficiently. Increased protein levels due to changing the *nagA*_*2*_ translational start codon from GTG to the more common ATG increased total activity. But even though total activity doubled, only around 10 % was found in the supernatant, showing that the endogenous NagA_2_ signal peptide supports secretion rather inefficiently.

Protein secretion has been studied for decades in *C. glutamicum* and recently a strain exhibiting potential as host for industrial-scale production of recombinant proteins has been commercialized as Corynex™. *C. glutamicum* is a favorable host for protein production and secretion, i.e. because it lacks extracellular proteolytic activities [51]. However, shortcomings such as its mycolic acid layer that in combination with the underlying peptidoglycan-arabinogalactan layer constitutes a second permeability barrier [52, 53] or an additional S-layer (present only in some strains) can hamper its utilization for protein production [54]. The strains used in this study are based on ATCC 13032, which lacks an S-layer [44]. *C. glutamicum* is not widely used for protein production, which may in part be due to variable yields depending on the target protein and on the target signal [42, 54–56].

The promoter and signal sequence of the *cspB* gene encoding PS2, the major protein secreted by the organism, have been used in many studies of heterologous protein secretion in *C. glutamicum* [43, 44, 57, 58]. A systematic screen of signal peptides for secretion of α-amylase from *Geobacillus stearothermophilus* in *C. glutamicum* showed up to 150-fold better secretion using signal peptides from e.g. genes *cg0955*, *cmt1* and *cmt4*, and *phoD* when compared to fusions with the PS2 secretion signal [43]. Conversely, secretion of endogenous NagA_2_ as well as heterologous ChiB fused to these signal peptides did not differ much in terms of overall secreted activity, utilizing different secretion signals (Fig. 1). Notably, signal peptides involved in the Sec pathway, (PS2, Cmt1 and Cmt4) or the Tat pathway (Cg0955 and PhoD) functioned similarly with NagA_2_ and ChiB (Fig. 1), whereas for other proteins, e.g. GFP, the translocation efficiency and/or final protein activity was strongly affected by the route of transport [59–61].

Despite of detection of GlcNAc units in culture supernatant, secretion of ChiB and NagA_2_ by *C. glutamicum* strain Δ*nanR* (pVWEx1-*nagE*) (pEKEx3-SP0955-*chiB*-SP0955-*nagA*_*2*_) was too low to support growth on colloidal chitin. Extracellular activities were never higher than 5 mU mL^−1^ (Fig. 1). This contrasts to recombinant *C. glutamicum* strains which are able to grow with starch as sole carbon source due to secretion of heterologous α-amylases [15, 57] due to about 100 fold higher α-amylase activities (400 to 650 mU mL^−1^).

A strategy for the hydrolysis of complex bio-polymers, alternative to the secretion of degrading enzymes, is their display on the cell surface, anchored to membrane integral proteins. One notable example for this approach is the heterologous expression of the α-amylase from *Streptococcus bovis* 148 (*amyA*), that allowed the growth with starch as carbon source when fused to *C. glutamicum* anchor proteins such as porins [62], the glutamate exporter NCgl1221 [63], or heterologous proteins such as PgsA from *B. subtilis* [16]. More recently, it was shown that the combined surface display in *C glutamicum* of endoglucanase [64] and β-glucosidase (BglA) from *Clostridium thermocellum*, anchored to the mechanosensitive channel Msc, enabled the saccharification of lignocellulosic material, leading to up to 6-fold increased reducing sugar generation when compared to secreted cellulases [65]. It remains to be established if surface display of N-acetylglucosaminidases and chitinases is superior to secretion of these enzymes. It may also be necessary to simultaneously overproduce chitinases with different mechanisms of action, e.g. combining an endo-chitinase, e.g. ChiC from *S. marcescens*, with a processive chitinase, e.g. ChiB from *S. marcescens* [24, 66, 67].

## Conclusion

*C. glutamicum* possesses β-N-acetylglucosaminidase. In the wild type, β-N-acetylglucosaminidase activity was too low to be detected. However, overproduction of the enzyme fused to TAT or Sec signal peptides led to secretion of active β-N-acetylglucosaminidase. The engineering of the signal peptide improved the secretion more than 60 folds as compared to the native sequence. NagZ of *B. subtilis* could be overproduced, but was not excreted efficiently, even when fused to *C. glutamicum* TAT or Sec signal peptides. Concomitant secretion of exochitinase ChiB from *S. marcescens* and endogenous NagA2 did not result in growth with colloidal chitin as sole or combined carbon source. This may indicate that either higher enzyme activities are needed or that additional enzymes such as processive chitinase or endochitinase activities may have to be overproduced.

## Methods

### Microorganisms, media, and cultivation conditions

The strains and plasmids used are listed in Table 5. The standard medium Luria–Bertani (LB) was used for *E. coli* [68], and brain heart infusion medium (BHI; Difco) was applied as complex medium for *C. glutamicum*. CgXII was used as minimal medium [69], supplemented with 100 mM N-acetyl-glucosamine and/or 100 mM N-acetyl-glucosamine, plus 10 g L^−1^ of colloidal chitin as carbon source. When appropriate, spectinomycin (100 mg L^−1^), kanamycin (25 mg L^−1^) and isopropyl-β-D-thiogalactopyranoside (IPTG, 0.1 mM) were added. *E. coli* was grown at 37 °C and *C. glutamicum* at 30 °C in 500 or 100 ml Erlenmeyer flask with 50 ml or 20 ml medium, respectively, on a rotary shaker at 120 rpm. Bacterial growth in liquid cultures was followed by measuring the optical density at 600 nm (OD_600_).

**Table 5:** Bacterial strains and plasmids used in this study

Strain or plasmid	Relevant characteristics	Reference
*E.coli*		
DH5α	F^−^ *thi* ^−^1 *end*A1 *hsdr17* (r^−^, m^−^) supE44 ΔlacU169 (Φ80*lac*ZΔM15) *rec*A1 *gyr*A96 *rel*A1	[75]
*C. glutamicum*		
WT	Wild-type strain ATCC 13032, auxotrophic for biotin	ATCC
Δ*nan*R	cg2936 deletion mutant of *C. glutamicum* WT	(Matano & Wendisch, unpublished)
Plasmids		
pVWEx1	KanR; *C. glutamicum*/*E. coli* shuttle vector (P*tac*, *lac*Iq; pHM1519, OriV*E.c.*)	[76]
pVWEx1-*nagE*	pVWEx1 carrying *nagE* (full gene) from *C. glycinophilum* ATCC 21341, with mutation (ATG start codon) and optimized RBS	[22]
pEKEx3	SpecR; *C. glutamicum*/*E. coli* shuttle vector (P*tac*, *lac*Iq; pBL1, OriV*E.c.*)	[3]
pEKEx3-*chiB*	pEKEx3carrying *chi*B gene from *S.marcescens* ATCC13880	This work
pEKEx3-*SP0955-chiB*	pEKEx3carrying *chi*B gene from *S.marcescens* ATCC13880, with signal sequence from *cg0955*	This work
pEKEx3-*nagA*_*2*_	pEKEx3carrying *nag*A_2_ gene from *C. glutamicum* WT	This work
pEKEx3-ATG-*nagA*_*2*_	pEKEx3carrying *nag*A_2_ gene from *C. glutamicum* WT, with mutation (ATG codon)	This work
pEKEx3- SP-less-*nagA*_*2*_	pEKEx3carrying *nag*A_2_ gene from *C. glutamicum* WT, without its own putative signal sequence.	This work
pEKEx3-*nagZ*	pEKEx3carrying *nag*Z gene from *B.subtilis* 168, codon optimized for expression in *E.coli*	This work
pEKEx3-*SP0955-nagZ*	pEKEx3carrying *nag*Z gene from *B.subtilis* 168, codon optimized for expression in *E.coli*., with signal sequence from *cg0955*	This work
pEKEx3-PS2-*nagZ*	pEKEx3carrying *nag*Z gene from *B.subtilis* 168, codon optimized for expression in *E.coli,* with signal sequence from PS2 (*csp*B) *C. glutamicum* ATCC 14067	This work
pEKEx3-PhoD-*nagZ*	pEKEx3carrying *nag*Z gene from *B.subtilis* 168, codon optimized for expression in *E.coli,* with signal sequence from *phod*	This work
pEKEx3-Cmt4-*nagZ*	pEKEx3carrying *nag*Z gene from *B.subtilis* 168, codon optimized for expression in *E.coli,* with signal sequence from *cmt4*	This work
pEKEx3-Cmt1-*nagZ*	pEKEx3carrying *nag*Z gene from *B.subtilis* 168, codon optimized for expression in *E.coli,* with signal sequence from *cmt1*	This work
pEKEx3-*SP0955-nagA*_*2*_	pEKEx3carrying *nag*A_2_ gene from *C. glutamicum* WT, with signal sequence from *cg0955*	This work
pEKEx3-PS2-*nagA*_*2*_	pEKEx3carrying *nag*A_2_ gene from *C. glutamicum* WT, with signal sequence from PS2 (*cspB*) *C. glutamicum* ATCC 14067	This work
pEKEx3-PhoD-*nagA*_*2*_	pEKEx3carrying *nag*A_2_ gene from *C. glutamicum* WT, with signal sequence from *phoD*	This work
pEKEx3-Cmt4-*nagA*_*2*_	pEKEx3carrying *nag*A_2_ gene from *C. glutamicum* WT, with signal sequence from *cmt4*	This work
pEKEx3-Cmt1-*nagA*_*2*_	pEKEx3carrying *nag*A_2_ gene from *C. glutamicum* WT, with signal sequence from *cmt1*	This work
pEKEx3-SP0955*-chiB-*SP0955*nagA*_*2*_	pEKEx3carrying *chi*B gene from *S.marcescens* ATCC13880, with signal sequence from *cg0955* and *nag*A_2_ gene from *C. glutamicum* WT, with signal sequence from *cg0955*	This work
pEKEx3-SP0955-*chiB*-PhoD-*nagA*_*2*_	pEKEx3carrying *chi*B gene from *S.marcescens* ATCC13880, with signal sequence from * cg0955* and *nag*A_2_ gene from *C. glutamicum* WT, with signal sequence from *phoD*	This work
pEKEx3- SP0955-*chiB*-Cmt4-*nagA*_*2*_	pEKEx3carrying *chi*B gene from *S.marcescens* ATCC13880, with signal sequence from *cg0955* and *nag*A_2_ gene from *C. glutamicum* WT, with signal sequence from *cmt4*	This work

### Preparation of colloidal chitin

A modified [70] method was used for colloidal chitin preparation. 5 g of chitin (Sigma) were added to 50 mL of concentrated hydrochloric acid. The mixture was incubated overnight at 4 °C while continuously stirring. Chitin was precipitated as colloidal suspension by the slow addition of deionized water and the chitin suspension was washed with deionized water until a neutral pH value was reached. The colloidal chitin obtained was resuspended in medium for growth assay in a final concentration of 10 g L^−1^.

### DNA preparation, manipulation and transformation

Plasmids were constructed in *E. coli* DH5α from PCR-generated fragments (KOD Hot Start DNA Polymerase; Novagen), using *C. glutamicum* ATCC 13032, *S. marcescens* ATCC 13880 genomic DNA as template. *C. glutamicum* genomic DNA was prepared as described [71]; *S. marcescens* ATCC 13880 (DSM 30121) genomic DNA was obtained by DSMZ German Collection of Microorganism and Cell Cultures (Braunschweig, Germany). A *nagZ* gene (GenBank Acc. No. CAB11942.1) from *B. subtilis* 168 (ATCC 23857) was synthesized in a codon optimized version for overexpression in *E. coli* (Genscript, USA). The oligonucleotides used in this study are listed in (Additional file 1: Table S1). *E. coli* was transformed by standard methods [68] and the plasmids were introduced by electroporation into *C. glutamicum* strains as described [69]. Transformants containing two compatible plasmids, here pVWEx1 and pEKEx3 and their derivatives, were selected by plating on agar plates containing kanamycin (25 μg/ml) and spectinomycin (100 μg/ml). Vector maps of pVWEx1-*nagE* and pEKEx3-SP0955-*chiB*-SP0955-*nagA2* are shown in Additional file 1: Figure S1. Transformants were analysed by PCR with appropriate primers; the absence of mutations in the cloned genes was verified by sequencing.

### Construction of plasmids and strains

For the construction of plasmids expressing *chiB*, *nagZ* and *nagA*_*2*_, with their own signal peptide or in fusion with *C. glutamicum* signal peptides, KOD PCR products were ligated into *BamHI*, *EcoRI* digested pEKEx3 via Gibson assembly [72]. The *chiB* gene was cloned into pEKEx3 in its native form and in fusion with a signal peptide from gene *cg0955* of *C. glutamicum* ATCC 13032 (hence SP0955). The gene *nagA*_*2*_ has been cloned in its native form, including its own signal peptide, with a mutation on the first triplet replacing the original GTG start codon with the ATG triplet, without its own putative signal sequence (hence SP-less- *nagA*_*2*_) and with its signal peptide replaced by secretion sequences from *C. glutamicum*, here named SP0955, PS2, PhoD, Cmt4 and Cmt1.

*B. subtilis nagZ* has been cloned with its own signal peptide or in a signal peptide-less form, in fusion with the above mentioned signal peptides SP0955, PS2, PhoD, Cmt4 and Cmt1.

### Glycol chitin preparation and dot blot chitinase assay

Glycol chitin was prepared by re-acetylation of glycol chitosan. 1 g of glycol chitosan (Sigma-Aldrich) was added to 20 mL of 10 % acetic acid and the mixture was incubated overnight while continuously stirring. 90 mL of methanol were slowly added and the solution was vacuum filtered on Buchner funnel. 1.5 mL of acetic anhydride were added to the filtrate and the solution was incubated 30 min at room temperature allowing the formation of a solid gel. The gel was cut into small pieces and homogenized in a waring blender. The homogenized gel was centrifuged and washed with methanol and resuspended a last time in 100 ml H_2_O to give a ca 1 % (w/v) solution of glycol chitin.

A dot blot assay on 12 % (v/v) acrylamide gel, with 50 mM TEA buffer (pH 7.0), containing 0.01 % (v/v) of glycol chitin was used for chitinolytic activity determination. Fractions of cell extract or supernatant from *C. glutamicum* WT (pEKEx3-*chiB*), *C. glutamicum* WT (pEKEx3) and Δ*nan*R (pEKEx3 SP0955-*chiB*) were spotted on the gel. After overnight incubation the gel was stained for 30 minutes with 0.01 % (w/v) Calcofluor White M2R (Sigma Aldrich, Germany) solution, destained and visualized by UV illumination [73]. Chitinolytic activity is revealed as dark halo on fluorescent background.

### Chitinase and N-acetylglucosaminidase activity assays

Aliquots from *C. glutamicum* BHI cultures were withdrawn during the exponential growth phase and cells where harvested by centrifugation (10 min, 3,200 x *g* and 4 °C). Supernatant and cell extract fractions from the aliquots were used for chitinase and N-acetylglucosaminidase activity quantification. Cells were disrupted by ultrasonic treatment (UP 200S; Dr. Hielscher GmbH, Teltow, Germany) with an amplitude of 50 % and a duty circle of 0.5 for 7 min, upon resuspension in 100 mM phosphate buffer (pH 7.0). The cell suspension was centrifuged for 1 h at 4 °C and 16,000 rpm and the soluble cell extract was recovered. Exo-chitinase and N-acetylglucosaminidase activity were determined by measuring the hydrolysis of p-nitrophenol from 4-Nitrophenyl N,N’-diacetyl-β-D-chitobioside and 4-Nitrophenyl N-acetyl-β-D-glucosaminide (Sigma Aldrich, Germany), respectively. The substrates were dissolved in 100 mM phosphate buffer (pH 7.0) solution at a concentration of 1 mg mL^−1^ and the activity was determined, following the chitinases assay protocol from Sigma Aldrich in 96 well plates, measuring the absorption at 405 nm of hydrolyzed p-nitrophenol upon addition of sodium carbonate stop solution. K_m_ values have been estimated with the Eadie Hofstee plot for NagA_2_ without its own signal peptide and as fusion protein with PhoD signal peptide, measuring the activity with different concentration of 4-Nitrophenyl N,N’-diacetyl-β-D-chitobioside in the cell extract fraction and in the supernatant, respectively.

### Quantification of GlcNAc via DNS colorimetric assay

GlcNAc released in the supernatant was determined spectrophotometrically by the Dinitrosalicylic acid (DNS) method following the protocol described by Miller [74] at 540 nm using 3 ml of culture supernatant. Concentrations of the released sugars were estimated using a standard calibration curve of GlcNAc (Sigma Aldrich, Germany) in a range of 0–2 mM.

## Additional file

Additional file 1:
**Table S1.** Oligonucleotides used in this study. **Figure S1** Plasmid maps of pVWEx1-*nagE* [A] and pEKEx3-SP0955-*chiB*-SP0955-*nagA2* [B]. Functional elements of the plasmid pVWEx1-*nagE* include antibiotic markers (Km, kanamycin), origin of replication (pHM1519), *nagE* (GlcNAc-specific PTS from *Corynebacterium glycinophilum* DSM45794). Functional elements of the plasmid pEKEx3-SP0955-*chiB*-SP0955-*nagA2* include relevant restriction sites, antibiotic markers (Spec, spectomycin), origin of replication (pBL1), tat (SP0955), *chiB* (chitinase B, *Serratia marcescens*), *nagA2 (*β-N-acetylglucosaminidase, *C. glutamicum). (DOCX 246 kb)*
