# Supplementary material for: Corynebacterium glutamicum possesses β-N-acetylglucosaminidase
Source: BMC Microbiol. 2016 Aug 5;16:177. doi: 10.1186/s12866-016-0795-3 (PMC4974736; doi:10.1186/s12866-016-0795-3)
Supplement: Additional file 1: — Table S1. Oligonucleotides used in this study. Figure S1 Plasmid maps of pVWEx1-nagE [A] and pEKEx3-SP0955-chiB-SP0955-nagA2 [B]. Functional elements of the plasmid pVWEx1-nagE include antibiotic markers (Km, kanamycin), origin of replication (pHM1519), nagE (GlcNAc-specific PTS from Corynebacterium glycinophilum DSM45794). Functional elements of the plasmid pEKEx3-SP0955-chiB-SP0955-nagA2 include relevant restriction sites, antibiotic markers (Spec, spectomycin), origin of replication (pBL1), tat (SP0955), chiB (chitinase B, Serratia marcescens), nagA2 (β-N-acetylglucosaminidase, C. glutamicum). (DOCX 246 kb) [file 12866_2016_795_MOESM1_ESM.docx]

**Supplementary Data to**

***Corynebacterium glutamicum* possesses β-N-acetylglucosaminidase**

**Christian Matano^1)^** ^†^**, Stephan Kolkenbrock^2)*^, Stefanie N. Hamer^2)^, Elvira Sgobba^1)^, Bruno M. Moerschbacher^2)^, Volker F. Wendisch^1)^**

^1)^ Chair of Genetics of Prokaryotes, Faculty of Biology & CeBiTec, Bielefeld University, 33501 Bielefeld, Germany

^2)^ Institute for Biology and Biotechnology of Plants, WWU Münster University, 48143 Münster, Germany

^†^ Current Address: GSK Vaccines S.r.l., Siena 53100, Italy.

^*^ Current address: altona Diagnostics GmbH, 22767 Hamburg, Germany

Email: Christian Matano – christian.x.matano@gsk.com; Stephan Kolkenbrock - [stephan.kolkenbrock@altona-diagnostics.com](mailto:stephan.kolkenbrock@altona-diagnostics.com); Stefanie N. Hamer - [s.hamer@biotec.rwth-aachen.de](mailto:s.hamer@biotec.rwth-aachen.de); Elvira Sgobba - [esgobba@cebitec.uni-bielefeld.de](mailto:esgobba@cebitec.uni-bielefeld.de); Bruno M. Moerschbacher - [moersch@uni-muenster.de](mailto:moersch@uni-muenster.de); Volker F. Wendisch - volker.wendisch@uni-bielefeld.de

**Table S1** Oligonucleotides used in this study.

| Name | Sequence (5´-3´)^a^ |
| --- | --- |
| pVWEx-fw | CATCATAACGGTTCTGGC |
| pVWEx-rv | ATCTTCTCTCATCCGCCA |
| ChiB_internF | AAGGCGCGGGTCAGCTCT |
| ChiB_internR | TGTGCGCTACCTCAGATA |
| nagZ-int1 | ATCGGCGTACGGTCATTCAG |
| nagZ-int2 | CGCGAAACAGCGACAGTACA |
| ChiB_F |  |
| ChiB_gibs_R | AAACGACGGCCAGTGAATTGGATCCGCTTACGCCACGCGGCCCACCTT |
| SP0955_F | CAGGTCGACTCTAGAGGATCGCCGTCGACG*GAAAGGAGGCCCTTCAG***ATG**CAAATAAACCGCCGAGG |
| SP0955-R-cross-chiB | CCGCTTTACGTGTGGACATTGCTCCAAGGGCGTTGGC |
| chiB_crosTAT_F | GGCCAACGCCCTTGGAGCA**ATG**TCCACACGTAAAGCGG |
| TAT-F-bam | CCTGCAGGTCGACTCTAGAGAGAGGATCCG*GAAAGGAGGCCCTTCAG***ATG**CAAATA |
| TAT-R-croschiB | ACAGCTTTACGGGTGGACATTGCTCCAAGGGCGTTGGC |
| rc1-NAGZ-frag-RBSF | GGTCGACTCTAGAGGATCGCCGGATCCG*GAAAGGAGGCCCTTCAG***ATG**CGCCCTGTCTTTCCGCTTATCCTTTCA |
| ps1-ps2-front-gibs | TGCAGGTCGACTCTAGAGGATCGCGGATCC*GAAAGGAGGCCCTTCAG***ATG**TTTAACAACCGTATCCGCACTGCAG CTCTC |
| ps2-ps_Rev | GGTCTCTTGAGCGAATGCTGGGATAGCTACGCCGGAAGCTGCGGTGGAGATTGCGATTGCACCAGCGAGAGCTGCAGTGC |
| ps3-nagA2-crosPS2_F | ATCCCAGCATTCGCTCAAGAGACC**ATG**GCAGAAGAG CCGGAACAG |
| ps5-nagZs-crosPS2_F | ATCCCAGCATTCGCTCAAGAGACC**ATG**TTTTTCGGCGCCCGTC |
| pho1-phoD_F-gibs | AGGTCGACTCTAGAGGATCCCCGCCGTCGACG*GAAAGGAGGCCCTTCAG***ATG**CCACAGTTAAGCAGACGC |
| pho2-phoD_R_gibs | CATTTGGCGTTCTTCAGCGCGT |
| pho3-nagA2-crospho_F | TGCACGCGCTGAAGAACGCCAA**ATG**GCAGAAGAGCCGGAACAG |
| pho4-nagZ_crospho_F | TGCACGCGCTGAAGAACGCCAA**ATG**TTTTTCGGCGCCCGT |
| cmt4-F-gibs | GTCGACTCTAGAGGATCCCCGCCGTCGACG*GAAAGGAGGCCCTTCAG***ATG**CGTAAAGGAATTTCCCGCGT |
| cmt4-R-gibs | CATAGAGTCTTGAGCTGCTGCGA |
| nagA2-cross-cmt4-F | GCATCGCAGCAGCTCAAGACTCT**ATG**GCAGAAGAGCCGGAA AG |
| nagZ-cross-cmt4-F | GCATCGCAGCAGCTCAAGACTCT**ATG**TTTTTCGGCGCCCGTC |
| cm1-cmt1-F-gibs | CAGTCGACTCTAGAGGATCCCCGCCGTCGACG*GAAAGGAGGCCCTTCAG***ATG**AAGCTTCTTCGCCGCATC |
| cm2-cmt1_rev | CATTACTTCGGCAGCGCCTGC |
| cm3-ngA2-crocmt1-F | CCACCGCAGGCGCTGCCGAAGTA**ATG**GCAGAAGAGC CGGAACAG |
| cm4-ngZ-cro-cmt1-F | CCACCGCAGGCGCTGCCGAAGTA**ATG**TTTTTCGGCG CCCGTC |
| tcm1-TAT-b-F | GCAGGTCGACTCTAGAGGATCGCCGTCGACG*GAAAGGAGGCCCTTCAG***ATG**CAAAT |
| tcm2-chiB_r | GAAGGGCCTCCTTTCCGTCGACGGCGGATCCGCTTACGCCACGCGGCCCA |
| tcm3-cmt1-F-croschib | TGGCGTAAGCGGATCCGCCGTCGACGGAAAGGAGGC CCTTCAG**ATG**AAGC |
| tcm4-nagA2_R-last | TAAAACGACGGCCAGTGAATTGGATCCGCTCACTTTTGTTCGAGACGTGTG |
| tcm5-nagZ-R-last | TAAAACGACGGCCAGTGAATTGGATCCGCTTACAGC GGACGACCCGTTT |
| agf2-nagA2Fatg-april | CTGCAGGTCGACTCTAGAGGATC*GAAAGGAGGCCCTTCAG***ATG**TGG |
| ChiB-R-gibson 2nd gene | GCTGAAGGTGGGCCGCGTGGCGTAAGCGTCGACG*GAAAGGAGGCCCTTCAG***ATG** |

a Restriction sites in the oligonucleotides are underlined, linker sequences for hybridization are depicted in grey,

artificial ribosome binding sites are shown in italics and start codons in bold.

**A**

**B**

**Figure S1 Plasmid maps of pVWEx1-*nagE* [A] and pEKEx3-SP0955-*chiB*-SP0955-*nagA2* [B].** Functional elements of the plasmid pVWEx1-*nagE* include antibiotic markers (Km, kanamycin), origin of replication (pHM1519), *nagE* (GlcNAc-specific PTS from *Corynebacterium glycinophilum* DSM45794). Functional elements of the plasmid pEKEx3-SP0955-*chiB*-SP0955-*nagA2* include relevant restriction sites, antibiotic markers (Spec, spectomycin), origin of replication (pBL1), tat (SP0955), *chiB* (chitinase B, *Serratia marcescens*), *nagA2 (*β-N-acetylglucosaminidase, *C. glutamicum).*
